# Therapeutic Applications of Extracellular Vesicles in Inflammatory Bowel Disease

**DOI:** 10.3390/ijms25020745

**Published:** 2024-01-06

**Authors:** Sang Hyun Kim, Bora Keum, Sooun Kwak, Junhyoung Byun, Jae Min Shin, Tae Hoon Kim

**Affiliations:** 1 Department of Internal Medicine, Korea University College of Medicine, Seoul 02841, Republic of Korea; snell1777@gmail.com (S.H.K.);; 2 Department of Otorhinolaryngology—Head & Neck Surgery, Korea University College of Medicine, Seoul 02841, Republic of Korea; 3 Mucosal Immunology Institute, Korea University College of Medicine, Seoul 02841, Republic of Korea

**Keywords:** extracellular vesicles, inflammatory bowel disease, drug delivery system

## Abstract

The treatment landscape for inflammatory bowel disease (IBD) has undergone substantial advancements with the introduction of biologics. However, a considerable number of patients either show an immediate lack of response or lose responsiveness over time, necessitating the development of innovative and effective treatment approaches. Extracellular vesicles (EVs) are small lipid bilayer-enclosed structures that facilitate cell-to-cell molecular transfer and are integral to the pathogenesis of IBD. They play pivotal roles in maintaining the integrity of the intestinal epithelial barrier and the expulsion of cellular metabolites. The potential use of EVs as drug carriers or therapeutic agents has opened up a plethora of clinical applications. This review investigates the creation and content of EVs, their role in IBD development, and advances in their isolation and analytical techniques. Furthermore, the therapeutic promise they hold for IBD is explored, along with the latest research on their roles as IBD drug delivery systems.

## 1. Introduction

Inflammatory bowel disease (IBD), encompassing both ulcerative colitis (UC) and Crohn’s disease (CD), is a chronic condition that causes inflammation within the digestive tract [1]. Prolonged inflammation, coupled with immune dysregulation, damages the gastrointestinal tract. Over the past decade, IBD has become a global disease with increasing incidence, especially among young adolescents [2,3]. Because the etiology and pathogenesis of IBD have not been accurately identified, it has polygenic and multifactorial properties [4]. IBD is characterised by impaired intestinal barrier integrity, microbial dysfunction, and a reduced ability to repair inflammatory damage in the gut [5]. A key element of immune regulation is the balance between immune suppression and stimulation, and a correct response to environmental triggers is the key element of immune regulation, which is disrupted in patients with IBD, leading to inflammation and impaired intestinal barrier integrity. Currently, the treatment for inflammatory bowel disease focuses on suppressing inflammation and healing the intestinal mucosa [6,7]. The advent of biological drugs that administer antibodies that inhibit various inflammatory mediators has improved the treatment environment for IBD [8]. However, many patients are initially unresponsive or gradually lose their response to biologics, with 10–20% of UC patients and 50% of CD patients requiring surgery within 10 years of diagnosis [9,10]. Moreover, patients treated with long-term anti-TNF-α agents have an increased risk of side effects such as opportunistic infections and malignancy [11]. Therefore, developing new treatments with suitable efficacy and safety for IBD patients is imperative.

Extracellular vesicles (EVs) are small spheres surrounded by a lipid layer that cells use to deliver small molecules to other cells. EVs are also involved in the pathogenesis of IBD [12] and play key roles in the regulation of intestinal epithelial barrier integrity and the export of cellular metabolites [13,14]. EVs can be found in numerous biological fluids, making them easily accessible for use in diagnosis, prognosis, and treatment. Various biomaterials derived from cells, from which vesicles are formed, are contained in EVs. In addition to the phospholipids that make up the cell membrane, EVs contain various types of surface and intracellular proteins and genetic materials [15]. Cells play an important role in molecular interactions via genetic materials transmitted through EVs. Moreover, EVs show potential as drug delivery substances or therapeutic agents, making their clinical applications wide. Compared to synthetic drug carriers, EVs have considerable advantages in terms of biocompatibility, immune tolerance, and non-toxicity [14].

In this review, we discussed the biogenesis and content of EVs and summarized their roles in IBD pathogenesis. Furthermore, we reviewed the latest advances in methodologies for EV isolation and analysis and summarized the therapeutic potential of EVs in IBD. Herein, we introduced recent research on the role of EVs as a drug delivery system for IBD.

## 2. Extracellular Vesicles

### 2.1. EV Biogenesis

**Figure 1:**
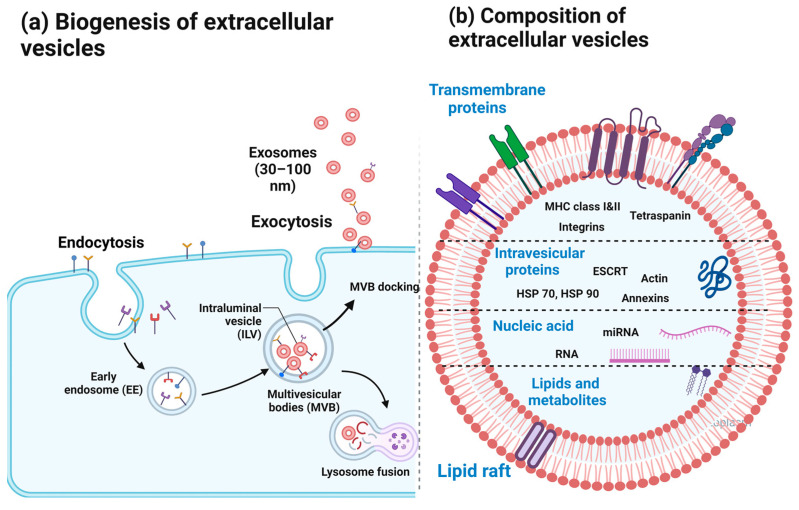
Biogenesis and molecular composition of exosomes. (**a**) Multivesicular bodies (MVBs) are created through the inward invagination of the endosomal-limiting membrane, resulting in an MVB composed of numerous intraluminal vesicles (ILVs). MVBs merge with either autophagosomes or lysosomes for degradation. However, some MVBs deviate from this course and are instead transported to the plasma membrane, where they dock. Subsequent exocytosis releases exosomes, which possess similar lipid bilayer orientation to the plasma membrane. (**b**) Diagrammatic representation of the membrane structure and contents of EVs. The membrane is composed of a lipid bilayer and incorporates various transmembrane proteins. Possible intravesicular proteins, lipids, and metabolites within the vesicle are also illustrated. EVs can transport nucleic acids like mRNAs of short length, and several non-coding RNAs. Partially created with BioRender.com, accessed on 13 December 2023.

EVs are classified into three groups based on their biogenesis process and size: exosomes (40–160 nm), microvesicles (100–1000 nm), and apoptotic bodies (>1000 nm) [16]. Microvesicles originate directly from the plasma membrane, whereas apoptotic bodies are generated by blebbing the plasma membrane during apoptosis [17]. Exosomes are EVs generated by intraluminal budding of the multivesicular body membrane (Figure 1) [16]. The plasma membrane’s inward folding forms cup-shaped structures, encompassing proteins that are soluble in the extracellular environment. This process is a precursor to the creation of early-sorting endosomes [18]. Early-sorting endosomes can mature into late-sorting endosomes and eventually produce intracellular multivesicular bodies (MVBs) [19,20]. These MVBs, which combine with the cell’s plasma membrane to expel their internal vesicles, are termed ‘exosomes’ [18].

### 2.2. EV Membrane Composition and Molecular Cargo

The membrane and cargo of EVs consist of proteins, nucleic acids, lipids, and metabolites (Figure 1). Proteins can be classified into intraluminal and transmembrane proteins [21,22]. EVs are typically rich in transmembrane proteins such as syndecans and tetraspanins. Syndecans and tetraspanins play important roles in exosome biogenesis. Moreover, tetraspanins are involved in cargo sorting and antigen presentation. Cell-specific proteins include MHC-I, MHC-II, and integrin expressed on the surfaces of EVs. Specific integrins vary depending on the cell type from which EVs are secreted. Common intraluminal proteins include the ESCRT machinery with related proteins, heat shock proteins, and cytoskeletal proteins [21]. The RNA species in EVs are diverse and mainly include mRNA and small regulatory RNA. When RNA present in EVs is delivered to target cells, it participates in gene expression to alter protein synthesis in the recipient cells [18,23]. For example, non-coding RNA miR-29a is transported by EVs and contributes to intestinal barrier integrity by downregulating aquaporin, NF-κB repressing factor, glutamine synthetase, and claudin-1 [24]. Lipids, including cholesterol, phospholipids, glycerophospholipids, diglycerides, arachidonic acid, sphingomyelin, and sphingolipids such as ceramide, are also important components of EVs [25]. Lipid bilayer membranes impart lipophilic properties to EVs and protect them from extracellular enzymes, allowing them to migrate easily within the intestinal wall without losing their biological activity [25].

## 3. Functional Roles of EVs in IBD Pathogenesis

EVs contain molecular particles from their parental cells, including RNAs and proteins, which facilitate their transport over long distances inside the body. EVs are involved in various immune responses in the body and play critical roles in the immune responses related to IBD. These include the modulation of immune responses, maintenance of intestinal barrier integrity, and shaping of the gut microbiota in IBD.

### 3.1. Immunomodulation

**Figure 2:**
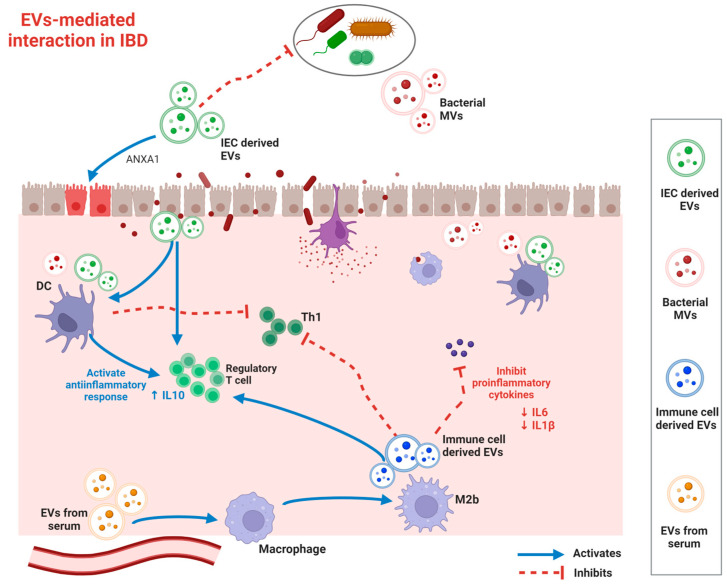
Role of extracellular vesicles (EVs) in the regulation of the intestinal microenvironment. EVs, derived from both the host and its commensal organisms, are instrumental in preserving intestinal homeostasis, maturing immune cells, and managing metabolic functions. However, in conditions like inflammatory bowel disease, the equilibrium between the host and its commensal organisms is disrupted. This imbalance is characterized by microbiota dysbiosis, an aberrant immune response, and a compromised intestinal barrier. Partially created with BioRender.com, accessed on 13 December 2023.

EVs are involved in immune regulation through the functional transfer of proteins, nucleotides, and other cargo between immune cells (Figure 2). An increase in innate immune cells, such as dendritic cells (DCs), macrophages, neutrophils, and T cells, has been observed in the active IBD intestinal environment. Macrophage regulation is crucial for the pathogenesis of IBD, and exosomes are involved in this process. Macrophages are classified into two types based on their function and activation: classically activated M1 macrophages and alternatively activated M2 macrophages. An imbalance between M1 and M2 macrophages is closely associated with inflammatory diseases, including IBD. EVs that polarize macrophages to the M1 type can worsen IBD [12,26]. Exosomes in visceral adipose tissue promote M1 macrophage polarization, leading to intestinal inflammation [27]. Systemically administered exosomes from human bone marrow mesenchymal stem cells (MSCs) considerably attenuate inflammation in IBD colitis models by polarizing M2b macrophages without causing intestinal fibrosis. Exosomes from polarized M2b macrophages increase the number of regulatory Treg cells and suppress proinflammatory cytokines, including IL-1β, IL-6, and IL-17A (Table 1) [26]. Regulating macrophage pyroptosis through exosomes can regulate inflammation in IBD [28]. Human umbilical cord mesenchymal stem cell (hucMSC)-derived exosomes carrying miR-203a-3p.2 attenuate colitis by inhibiting CASP11/4-induced macrophage pyroptosis [29].

T-cells are key members of the adaptive immune response and play crucial roles in IBD pathogenesis. Regulatory T cells (Tregs) and T-helper 17 (Th17) cells are critical for immune homeostasis and play crucial roles in IBD [30]. An imbalance between Th17 and Treg cells is a major cause of intestinal tolerance failure and contributes to IBD [31]. MSC-Exos alleviate IBD colitis by normalizing the Th17/Treg cell balance. Olfactory ecto-mesenchymal stem cell-derived exosomes exerted immunosuppressive effects by inhibiting Th1 and Th17 cell differentiation and promoting Treg cell induction in a murine colitis model [32]. EVs derived from Tregs induce the transformation of other T cells into the Treg phenotype (Table 1). In addition, EVs isolated from breast milk stimulated the development and proliferation of Tregs in a dose-dependent manner [33].

DCs are antigen-presenting cells (APCs) capable of activating naive T cells, which facilitate IBD pathogenesis. DC-derived EVs influence IBD progress via immunomodulation. Exosomes from DCs (DC-Exos) treated with IL-10 upregulate the levels of Tregs in the colonic lamina propria and attenuate colitis in a mouse model [34]. Moreover, exosomal miRNAs modulate the immune system in IBD by regulating intestinal immune cell responses. Exosomal miR146a and miR155 enter recipient DCs and mediate the suppression of their target genes [35].

### 3.2. Intestinal Barrier Modulation

The impairment of the intestinal barrier is a significant contributor to the development and progression of inflammatory bowel disease (IBD). EVs help repair damaged intestinal epithelial barriers and recover gastrointestinal function in patients with IBD. Extracellular vesicles (EVs) have the ability to influence the function of different types of intestinal epithelial cells (IECs) and control the stability of junctional complexes among IECs (Table 1) [36]. DC exosomes recover intestinal barrier function in DSS-induced colitis via exosomal miR-146b, activating the NF-kB signaling pathway [37]. The overexpression of miR-146b activated the NF-κB pathway, improved epithelial barrier function, and relieved intestinal inflammation in DSS-induced colitis mice. miR-146b improves intestinal inflammation by upregulating NF-κB as a result of the decreased expression of siah2, which ubiquitinates TRAF proteins [37]. LncRNA NEAT1 regulates the intestinal epithelial barrier and inhibits inflammatory responses in IBD [38]. Exosomal miR-223 destroys intestinal barrier function and increases intestinal epithelial permeability by inhibiting the TJ-related protein claudin-8 in IECs [39]. IEC accelerates the recovery of intestinal wall function by secreting EVs containing annexin A1 (ANXA1), a protein that activates wound recovery in patients [40]. IEC also releases EVs containing high levels of TGF-β1 to restore immune balance in the inflamed bowel.

### 3.3. Gut Microbiome Modulation

EVs in host cells participate in the network between the host and intestinal microbes, serving a crucial function in microbial reconstruction. Recent studies have found pathways by which altered gut microbiota may affect the development of IBD [41]. For instance, the activity of TLR 2/6 in probiotics is significantly reduced by EVs from TLR 2-deficient mouse sera, leading to microbial dysbiosis [42]. *Akkermansia muciniphila*-derived EVs can enhance epithelial barrier function and decrease the production of IL-6 by IECs [43]. *Lactobacillus plantarum* Q7-derived EVs reduce the pro-inflammatory bacteria Proteobacteria and increase the anti-inflammatory bacteria Bifidobacteria and Muribaculaceae, attenuating colitis [44]. *Clostridium butyricum*-derived EVs also play a role in restoring gut barrier function, normalizing bacterial dysbiosis, and restoring miR-199a-3p expression [45]. Exosomal nucleic acids participate in microbial modulation. miRNAs such as miR-515-5p and miR-1226-5p can enter *Fusobacterium nucleatum* and *Escherichia coli* to regulate bacterial gene transcripts and affect bacterial growth [46].

## 4. Current Methodologies for EV Isolation and Analysis

Developing sensitive, accurate, and standardized bioassays that can determine the composition and molecular profile of EVs in clinical samples is imperative for the rapid clinical application of EVs [47]. EVs are novel targets that are different from current analysis targets and have unique size characteristics; they are much smaller than cells but larger than proteins [20]. Technical problems often occur when conventional analysis methods are used and inaccurate results are obtained. Therefore, the development of new isolation and analysis techniques that consider the characteristics of EVs is an important goal. Recently, various techniques for exosome isolation and analysis have been developed. Therefore, understanding the characteristics, strengths, and weaknesses of each technology type is essential. Adopting and using appropriate technologies for each environment is important in research and clinical applications. Because various separation technology methods have distinct advantages and disadvantages, understanding their characteristics and selecting an optimal separation technology that matches the given sample characteristics are important for obtaining highly reproducible results.

### 4.1. EV Isolation

For clinical applications in diagnosis and treatment, it is necessary to isolate EVs first. The introduction of standard methods with the highest purification quality and lowest impact on EV components remains a potential challenge. Exosome isolation is usually based on physicochemical properties such as density, size, and mass, as well as affinity interactions with specific proteins.

Ultracentrifugation (UC) has been introduced as the gold standard for EV isolation [48]. However, some obstacles limit the clinical application of UC, including expensive equipment, training, large input samples, and labor-intensive and long run times [49]. The EV structure may change because of the high centrifugal force, which may affect downstream analysis. EVs were isolated using density gradient ultracentrifugation (DGC) based on their size, mass, and density using a pre-constructed density gradient medium. Although this method maintains the structural integrity of EVs, it is time-consuming and laborious [47].

Various methods, such as ultrafiltration and size-exclusion chromatography, have been applied to separate exosomes using size-based separation techniques. Owing to their fast separation speed and high efficiency, these two techniques are the most widely used, and several commercial products that use these methods are being released. However, separating exosomes of different sizes is difficult because of their low resolution. To overcome this, symmetric flow field-flow fractionation (AF4) was recently developed [50]. Using this method, large exosome vesicles (90–120 nm) can be separated from small exosome vesicles (60–80 nm) [51].

The precipitation separation method was commercialized early owing to its simplicity and high separation efficiency. Because aqueous polymers reduce the hydration of EVs and cause precipitation, the precipitated EV products can be easily isolated with low centrifugal forces. However, co-precipitation protein interference is caused by nonspecific interactions between the polymers and proteins.

The immunoaffinity capture-based technique is a separation method in which antibodies are fixed to surfaces such as microfluidic chips, nanostructures, and beads, and specific proteins present on the exosome surface are targeted and captured [47]. This method can be used to selectively separate and concentrate a very small group of exosomes from specific clinical samples, such as those for cancer diagnosis.

### 4.2. EV Analyzation

Exosome characterization technologies are currently being developed to comprehensively understand the morphological and physicochemical characteristics of exosomes. Therefore, developing various characterization techniques to analyze different subsets of EVs is necessary. Dynamic light scattering (DLS) and nanoparticle tracking analysis (NTA) are used as analytical methods to determine the size distribution of exosomes [52]. Western blotting (WB), enzyme-linked immunosorbent assays (ELISA), and flow cytometry are capable of determining the surface proteins of exosomes [52]. Protein analysis using WB or ELISA methods has limitations in that it requires a large number of samples and is time-consuming. Current protein analysis in EVs without strong amplification strategies has technical limitations. For instance, applying flow cytometry to extracellular vesicle analysis is challenging and requires careful consideration [52]. This is because the rapid movement of EVs, which are much smaller than the cell size, produces a weak signal in flow cytometry.

Therefore, new analytical technologies have been developed and proposed. Among these, technologies that are close to commercialization are based on nanoplasmonics [53]. Plasmonic sensors are optical sensors that use metal films or nanostructures. This technique has been widely used to detect analytes and characterize their molecular interactions over the past 30 years. The detection range (10–300 nm) of the plasmonic sensor is similar to the size of the exosomes (50–200 nm) and has a very high sensitivity for exosome detection.

## 5. EVs as Natural Therapeutic Agents for IBD

**Figure 3:**
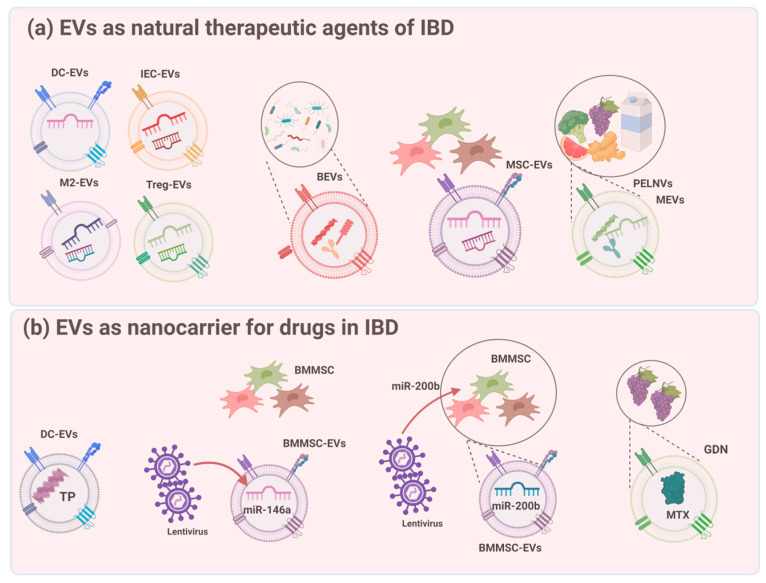
Therapeutic potential of EVs in IBD. (**a**) Natural exosomes without any genetic/chemical modification. (**b**) Loading therapeutic agent into exosomes through exosome engineering. Partially created with BioRender.com, accessed on 13 December 2023.

Natural EVs are considered new potential treatments for IBD. EVs originate from various sources, such as immune cells, IECs, the gut microbiota, various MSCs, and ingesta (Figure 3). The advantages of using EVs as direct therapy for IBD include safety, effectiveness, and cost-efficiency. Given that research in the field of EVs is still in its early stages, there are a limited number of studies that have conducted direct one-to-one comparisons of their efficacy in animal models of IBD [54].

### 5.1. Stem Cell-Derived EVs

EVs from stem cells play important roles in immune regulation, self-renewal, expansion, and damage repair (Table 1) [55]. Treatment with SC-EVs is more convenient in terms of transportation and storage because it prevents various side effects that may be caused by stem cell transplantation. Diverse MSCs are the fundamental sources of EVs in humans, including bone marrow MSCs (BMMSCs) [56], human adipose MSCs (hADSCs) [57], olfactory ecto-MSCs (OE-MSCs) [32], and human placental MSCs (hP-MSCs) [58]. MSC-EVs exert therapeutic effects against IBD mainly through modulation of the intestinal barrier, immune response, and intestinal microbiota (Figure 3). MSC-EVs can maintain barrier integrity by reducing IEC apoptosis, reversing goblet cell loss and microvilli and TJ function, and activating IEC and ISC proliferation [57]. MSC-EVs are capable of adjusting pro-inflammatory cytokines (e.g., TNF-α, IL-1β, IL-6, IL-12, IL-17, and IFN-γ) and anti-inflammatory cytokines (e.g., IL-4, IL-13, IL-10, and TGF-β) [32,58,59]. Moreover, MSC-EVs mediate immune cells such as Treg cells, M1/M2 macrophages, and Th1/Th17 subpopulations [32]. MSC-EVs inhibits colonic macrophages, downregulates inflammatory cytokine levels, and inhibits the NF-kB signaling pathway for treating colitis. MSC-EVs can improve the gut microbiota composition by significantly restoring the structure of OTUs (operational taxonomic units) and colitis-induced reduction in α-diversity, increasing the abundance of ‘healthy’ bacteria, decreasing disease-associated bacteria, decreasing detrimental functions, and enhancing other vital cellular functions [60,61]. The inflammation-modulating effects of hucMSC-EVs in IBD have also attracted attention. HucMSC exosomes modulate inflammation in DSS-induced colitis by regulating ubiquitin modification levels, neddylation inhibition by exosomal miR-326, and IL-7 expression in macrophages [62,63]. HucMSC-EVs are also involved in intestinal fibrosis prevention in IBD colitis models.

### 5.2. EVs from IECs and Immune Cells

The protective effect of IEC-EVs on the intestinal mucosa is well known (Table 1). Endogenous aANXA1 is released as a component of EVs derived from the intestinal epithelial cells. Leoni et al. showed that ANXA1-containing EVs accelerate the recovery of epithelial barrier dysfunction in IBD [40]. Exosomes extracted from various M2 macrophage subtypes can be used for treatment. Exosomes from M2b macrophages may exert a protective effect by increasing the number of Treg cells and IL-4 levels in the spleen [26]. DC-Exos treated with *Schistosoma japonicum*-soluble egg antigen also significantly improved DSS-induced acute colitis in mice [64]. These EVs reduced pro-inflammatory cytokine expression and improved all the analyzed colitis parameters.

### 5.3. EVs from the Microbiota

FMT is a popular treatment that uses gut flora reconstitution to restore the normal microbiome; however, people with IBD have higher risks [65]. Therefore, Bacterial EVs (BEVs) have been considered as potential treatments (Figure 3). EVs derived from *Clostridium butyricum* attenuated murine DSS-induced colitis by remodeling the composition of the gut microbiota and repolarizing M2 macrophages [60]. EVs derived from the probiotic Lactobacillus plantarum Q7 also improved the dysregulation of the gut microbiota and promoted the diversity of the gut microbiota in IBD mice [44]. EVs originating from *Akkermansia muciniphila* augment the expression and functionality of tight junctions, thereby reinstating the intestinal permeability of Caco-2 cells (Table 1) [43].

### 5.4. EVs from the Ingesta

Recently, EVs derived from the ingesta have received considerable attention because of their availability, safety, and favorable cost [66]. Milk is a primary source of ingested EVs for IBD treatment. Milk-derived EVs (MEVs) play an essential role in the maturation of the gastrointestinal tract. In a study by Tong et al. [67], milk-derived extracellular vesicles (mEVs) derived from bovine and human breast milk exerted similar protective effects on epithelial tight junction functionality in vitro. The oral administration of mEVs restores gut barrier integrity at multiple levels, including the mucus, epithelial, and immune barriers. Incubation with mEVs upregulated the expression of tight junction proteins, ZO-1, and Occludin in Caco-2 cells. They can also restore disturbed gut microbiota and enhance the intestinal barrier by decreasing matrix metallopeptidase 9 (MMP9) and increasing mucoprotein 2 (MUC2) [68,69]. Daily consumed food items like fruits and vegetables, as well as exosome-like nanoparticles derived from them, also engage with the intestinal mucosa through osmotic processes (Figure 3). Wang et al. showed that grapefruit-derived exosome-like vesicles (GDNs) were selectively absorbed by macrophages in the intestine, which led to an increase in heme oxygenase-1 levels and a decrease in IL-1 and TNF-α (Table 1) [70]. Additionally, both grapes (GELNs) and broccoli (BDNs) release exosome-like nanoparticles that serve as therapeutic aids in alleviating DSS-induced colitis (Table 1). These nanoparticles function by triggering intestinal tissue remodeling mediated by stem cells and stimulating DC amp-activated protein kinase [71,72]. Nanoparticles derived from ginger can be assimilated by IECs and macrophages, leading to fortification of the intestinal barrier and restoration of the gut microbiota [73]. The oral administration of turmeric-derived nanovesicles exhibited excellent anti-inflammatory efficacy in IBD by regulating the intestinal microbiota, repairing the damaged intestinal barrier, and transforming M1 macrophages into M2 macrophages [74].

## 6. EVs as Nanocarriers for Drugs in IBD

Efforts to develop nanoparticle-based drug delivery technologies have been ongoing over the past decade. However, nanoparticles are removed by the mononuclear phagocyte system during the blood cycle; eventually, less than 1% of the drug dose reaches the target tumor. In contrast, exosomes have the potential to act as highly efficient drug carriers. In particular, exosomes can be successful platforms for various drug delivery systems because of their ability to mediate cellular communication. Exosomes can be modified to exhibit the desired targeting ability through parent cell modification and can be loaded with various therapeutic agents. Exosomes can combine the advantages of existing synthetic nanoparticle-based drug carriers and cell-mediated delivery methods while complementing the shortcomings of each method (Figure 3).

### 6.1. Cargo Loading Techniques

**Figure 4:**
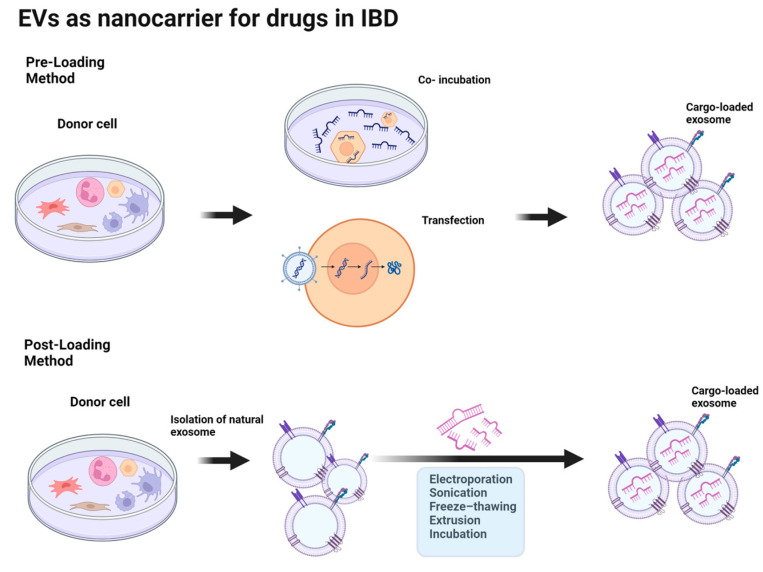
Drug loading methods for exosomes. Partially created with BioRender.com, accessed on 13 December 2023.

The primary methods through which therapeutic agents are integrated into exosomes are pre-loading (indirect loading) and post-loading (direct loading) (Figure 4) [75]. In the pre-loading technique, drugs are loaded into parental cells before exosome isolation, allowing them to be encapsulated within exosomes during their natural biogenesis [76]. This method involves transfecting the target gene to produce targeted exosomes or modifying parental cells by co-incubating them with the cargo. A major consideration of this method is the use of parent cells to obtain exosomes. To obtain a large number of exosomes, a large number of parental cells are required, and the method for increasing the production yield of exosomes is also important. The pre-loading method allows for the continuous generation of exosomes loaded with cargo while preserving membrane integrity [77] and has high stability and low immunogenicity. However, the parental cells may be potentially toxic because of the transfection agents. Another disadvantage is its low efficiency compared to that of electroporation and ultrasonic loading technologies [75].

The post-loading method incorporates the drug into pre-isolated natural exosomes. The post-loading method provides improved efficiency compared to the pre-loading method, as it allows for more effective control of the loading capacity. The post-loading method includes two distinct strategies: passive loading and active loading (Table 2). Passive loading using the incubation method allows the drug to passively diffuse into exosomes owing to its physical properties. This method is simple and nondestructive for maintaining exosome integrity. However, this method is mainly useful for hydrophobic drugs that interact with the lipid surfaces of exosomes, which is a potential limitation [78]. The lipid bilayer structure of the vesicle membrane facilitates the loading of hydrophobic drugs such as curcumin and paclitaxel into exosomes through incubation. Recently, considerable progress has been made in the development of active cargo-loading strategies. Various methods have been proposed, including electroporation, sonication, freeze–thawing, and extrusion, which can efficiently load cargo molecules by inducing temporary pores or mechanical stress in exosome membranes [75]. These methods enable precise control of the loading process to optimize the loading conditions and achieve the desired level of cargo loading. Table 2 summarizes the advantages and disadvantages of these methods.

Nevertheless, depending on the employed methodology, certain limitations and risks need to be taken into account. These may include exosome aggregation and the possibility of membrane damage arising from variations in pH, physical forces, and surfactants. Selecting the optimal cargo-loading method according to the cargo content and treatment purpose is crucial.

### 6.2. Application of Exosomes as Nanocarriers in IBD

Recently, MSCs have gained considerable attention as promising nanocarriers for IBD treatment. MSC-derived exosomes deliver cytokines, miRNAs, and external cargo to the immune microenvironment to regulate immune effector cells. miR-146a significantly attenuated drug-induced colitis in a mouse model after lentiviral transfection into BMMSC-EVs. miR-200b, after being transfected into BMMSCs with lentivirus, could be transferred to IEC-6 cells by being packaged in EVs, improving colon fibrosis. Moreover, DCs can regulate intestinal immunity through exosomes containing various antigenic peptides. Various anti-inflammatory factors (including IL-10, IL4, and TGF-β1) interacted with DCs to generate various immunomodulatory exosome subtypes. Exosomes derived from the TGF-β1 gene-modified bone marrow DC (TGF-β1-EXO) have inhibited Th17 responses and upregulated regulatory T cells, playing a protective role during IBD development in a murine model. Exosomes derived from IL-10-treated bone marrow-derived DCs reduced the severity of drug-induced colitis in mice. Alexander et al. found that endogenous miR-155 and miR-146a, two critical miRNAs that regulate inflammation, are released from dendritic cells within exosomes and are subsequently taken up by recipient dendritic cells. Exogenous miRNAs mediate target gene repression and can reprogram the cellular response to endotoxins, where exosome-delivered miR-155 is enhanced, whereas miR-146a reduces inflammatory gene expression.

Exosomes secreted by M2 macrophages are involved in the resolution of inflammation and intestinal repair. M2 macrophage-derived exosomal miR-590-3p reduces inflammatory signals and promotes epithelial regeneration by targeting LATS1 and subsequently activating YAP/β-catenin-regulated transcription. The role of M2 macrophage-derived EVs (M2-EVs) in UC inflammation has also been investigated. M2 macrophages were transfected with lentiviral vectors overexpressing LncRNA maternally expressed 3 (MEG3). High expression of MEG3 in EV was observed after isolation. M2-EVs carrying MEG3 enhanced UC cell viability and reduced inflammatory responses via the miR-20b-5p/CREB1 axis, thereby attenuating inflammation in a murine IBD model.

## 7. Conclusions and Future Perspectives

In the current review, we summarized the roles of EVs of different origins in the pathogenesis and progression of IBD, as well as their potential in clinical diagnosis and therapy. In addition, we introduced recent studies on the role of EVs as drug delivery systems in IBD. Exosomes are being actively researched in various fields, including isolation and analysis technologies and the development of diagnostic and treatment methods. However, because the history of exosome research is limited, a standardized method has not yet been developed. Exosome-mediated treatment technologies have received attention in various fields, and several early clinical trials have been conducted.

However, compared to current drug delivery systems that use nanoparticles or liposomes, this is in its early stages, and its strengths and weaknesses have been identified. More research is needed on the in vivo distribution of exosomes, their mechanisms, and their potential toxicity when administered in vivo.

The production yield of exosomes depends on the secretion capacity of the cell, the complexity and cost of large-scale cell culture, and the time required for exosome production. Industrial production is difficult because of low yields, which is an obstacle that cannot be ignored. As previously mentioned, selecting and modifying donor cells for exosome production and developing new methods to maximize the uniform production of exosomes are the needs of the hour.

Additionally, exosomes have limited cargo transport efficiency. Exosomes inherently carry large amounts of natural components that significantly complicate and limit their cargo loads. Therefore, developing exosome engineering techniques for improved loading capacities is necessary.

Another disadvantage is that the quality control of exosomes is difficult. Exosomes are highly heterogeneous, even when produced by a single cell type. Because of the lack of sensitive, high-efficiency exosome analysis methods, separating homogeneous exosomes from heterogeneous exosome groups is technically challenging.

Currently, strategies to precisely control the content of exosomes are lacking, which hinders the development of treatment technologies using exosomes. However, if new creative approaches are developed to overcome these shortcomings, a new era of IBD treatment will emerge. The high biocompatibility of exosomes provides greater opportunities for clinicians to develop safe and targeted therapies for IBD treatment and management.

## Figures and Tables

**Table 1:** Overview of the EVs suppressing IBD pathogenesis.

EV Sources	EV Types	Effects on Suppressing IBD Pathogenesis	
Stem Cells Derived EVs			
hucMSCs	Exosomes	Protect intestinal barrier	- Increase tight junction (TJ) expression - Restore goblet cells - Improve permeability of the intestinal mucosa
		Suppress inflammation	- Downregulate proinflammatory/anti-inflammatory cytokines - Regulate the reaction of Th2 and Th17 cells
		Improve gut microbiota composition	- Enhance the presence of beneficial microbes - Reduce the prevalence of bacteria associated with diseases.
hP-MSCs	Exosomes	Suppress inflammation	- Balance anti-inflammatory cytokines and pro-inflammatory cytokines
		Suppress oxidative stress	- Reduce the function of myeloperoxidase (MPO) and reactive oxygen species (ROS)
		Protect the intestinal barrier	- Minimize apoptosis in intestinal epithelial cells (IECs) - Stimulate the proliferation of both IECs and intestinal stem cells (ISCs). - Support the preservation of TJs
BMMSCs	Exosomes	Suppress inflammation	- Enable M2b polarization and enhance resistance to inflammation in macrophages within the colon
EVs from IECs and immune cells			
Treg cells	Exosomes	Protect the intestinal barrier	- Stimulate the growth of IECs and suppress their programmed cell death through miR-195a-3p
M2 macrophages	Exosomes	Protect the intestinal barrier Suppress inflammation	- Enhance IEC proliferation via epithelial YAP - Promote wound healing of IECs by exosomal miR-590-3p - Inhibit the induction of pro-inflammatory cytokines - Increase Treg cells in spleens
IECs	Exosomes	Recover the epithelial barrier	- Exosomal ANXA1 initiates the activation of wound healing pathways through FPR signaling
IECs	Exosomes	Suppress inflammation	- Induce immunosuppressive DCs and Treg cells
EVs from microbiota			
*Lactobacillus plantarum, Clostridium butyricum*	CMVs Exosomes	Suppress Inflammation Improve gut microbiota	- Promote the infiltration of M2 macrophages into the colon and inhibit M1 macrophage polarization - Diminish neutrophil infiltration - Balance pro-inflammatory and immunoregulatory cytokines - Reduce Th1 and Th17, and increase Th2 and Treg cells in colon - Reduce pro-inflammatory bacteria and increase anti-inflammatory bacteria
*Akkermansia muciniphila*	OMVs	Recover the epithelial barrier	- Increase TJ expression - Increase the gut permeability of Caco-2 cells
EVs from ingesta			
GELNs, GDNPs-2	PELNVs	Enhance intestinal barrier	- Remodulate Lgr5+ ISCs via β-catenin-mediated signaling pathways - Protect IECs
GDNs	PELNVs	Suppress inflammation	- Upregulate heme oxygenase-1 (HO-1) expression - Inhibit IL-1β production and TNF-α in intestinal macrophages
BDNs	PELNVs	Suppress inflammation	- Prevent DC activation and induce tolerant DCs via AMPK activation in DCs
EVs from parasites			
Trichinella spiralis	Exosomes	Suppress inflammation	- Reduce neutrophil infiltration - Balance pro-inflammatory and immunoregulatory cytokines

Abbreviations: OMVs, outer membrane vesicles; CMVs, cytoplasmic membrane vesicles; PELNVs, plant exosome-like nanovesicles; GELNs, grape exosome-like nanoparticles; GDNPs-2, ginger-derived nanoparticles; GDNs, grapefruit-derived nanovesicles; BDNs, broccoli-derived nanoparticles.

**Table 2:** Outline of various exosome cargo loading techniques.

Engineering Strategy	Type of Strategy	Types of Cargos	Advantages	Disadvantages
Passive cargo loading	Incubation	Drugs: paclitaxel [79], doxorubicin [80], curcumin [81], celastrol [82], porphyrins [83] Nucleic acids: siRNA, microRNA, Nanomaterials (MIL-88A), Fe_3_O_4_.	Quick and simple, no effects on exosome integrity	Low efficiency, more effective for hydrophobic compounds
Active cargo loading	Electroporation	Drugs: paclitaxel [84], doxorubicin [85] Nucleic acids: siRNA, shRNA [86], mRNA [87] Nanomaterials: PMA/Au-BSA@Ce6 [88], hollow Au nanoparticles [89]	Simple and quick	Destroying the integrity of the membrane structure, reducing the loading efficiency, low loading rate
	Sonication	Drugs: paclitaxel [84], doxorubicin [90], gemcitabine Enzymes: catalase [91] Nanomaterials: hollow Au nanoparticles [89]	Loading of hydrophobic and hydrophilic compounds	Exosome aggregation, compromised membrane integrity, changes in size
	Freeze–thaw cycles	Enzymes: catalase [91]	Simple and quick	Low drug loading capacity, exosome aggregation, changes in size
	Extrusion	Drugs: doxorubicin [83], 5-fu, gemcitabine, carboplatin [92] Enzymes: catalase [91]	Higher drug loading capacity	Damage to the plasma membrane structure
	Membrane permeabilizers	Drugs: porphyrins [83]	Relatively effective drug loading strategy	Immunogenicity, additional purification steps

Abbreviations: Fe_3_O_4_, ferrosoferric oxide; miRNA, microRNA; mRNA, messenger RNA; PMA/Au-BSA@Ce6, gold nanoparticles coated with an amphiphilic polymer and coupled with chlorin 6 containing bovine serum albumin; shRNA, short hairpin RNA; siRNA, short interfering RNA.

## Data Availability

The datasets in this study are available from the corresponding author on reasonable request.
